# Dynamic mRNA Expression Analysis of the Secondary Palatal Morphogenesis in Miniature Pigs

**DOI:** 10.3390/ijms20174284

**Published:** 2019-09-01

**Authors:** Jia Liu, Jing Chen, Dong Yuan, Lindong Sun, Zhipeng Fan, Songlin Wang, Juan Du

**Affiliations:** 1 Department of geriatric dentistry, Capital Medical University School of Stomatology, Tiantan Xili No. 4, Beijing 100050, China; 2 Laboratory of Molecular Signaling and Stem Cells Therapy, Molecular Laboratory for Gene Therapy and Tooth Regeneration, Beijing Key Laboratory of Tooth Regeneration and Function Reconstruction, Capital Medical University School of Stomatology, Tiantan Xili No. 4, Beijing 100050, China; 3 Department of Biochemistry and Molecular Biology, Capital Medical University School of Basic Medical Sciences, You An Men Wai Xi Tou Tiao No. 10, Beijing 100069, China

**Keywords:** gene expression profile, palate development, cleft lip/palate, miniature pig

## Abstract

Normal mammalian palatogenesis is a complex process that requires the occurrence of a tightly regulated series of specific and sequentially regulated cellular events. Cleft lip/palate (CLP), the most frequent craniofacial malformation birth defects, may occur if any of these events undergo abnormal interference. Such defects not only affect the patients, but also pose a financial risk for the families. In our recent study, the miniature pig was shown to be a valuable alternative large animal model for exploring human palate development by histology. However, few reports exist in the literature to document gene expression and function during swine palatogenesis. To better understand the genetic regulation of palate development, an mRNA expression profiling analysis was performed on miniature pigs, *Sus scrofa*. Five key developmental stages of miniature pigs from embryonic days (E) 30–50 were selected for transcriptome sequencing. Gene expression profiles in different palate development stages of miniature pigs were identified. Nine hundred twenty significant differentially expressed genes were identified, and the functional characteristics of these genes were determined by gene ontology (GO) function and Kyoto Encyclopedia of Genes and Genomes (KEGG) pathway analysis. Some of these genes were associated with HH (hedgehog), WNT (wingless-type mouse mammary tumor virus integration site family), and MAPK (mitogen-activated protein kinase) signaling, etc., which were shown in the literature to affect palate development, while some genes, such as *HIP* (hedgehog interacting protein), *WNT16*, *MAPK10*, and *LAMC2* (laminin subunit gamma 2), were additions to the current understanding of palate development. The present study provided a comprehensive analysis for understanding the dynamic gene regulation during palate development and provided potential ideas and resources to further study normal palate development and the etiology of cleft palate.

## 1. Introduction

The most frequent birth defects causing craniofacial abnormalities are congenital cleft lip/palate (CLP). The majority of CLP (70%) are regarded as nonsyndromic, with the clefts occurring without other anomalies. Children are affected by considerable morbidity, families are burdened with substantial financial risk, and a concomitant social burden may be caused by CLP. Patients with CLP may experience problems with feeding, speaking, and hearing and undergo subsequent treatment from surgery, dental treatment, and speech training to psychosocial intervention [1,2,3]. As CLP is considered a complex etiology with both genetic and environmental factors, occurs in the early stage of development, and has modest recurrence rates, specific etiologic factors are difficult to identify. Deeper insights into the pathogenesis of CLP may be explored by an integration of candidate genes and genome-wide studies and analyses of epidemiologic and animal models.

Mammalian palatogenesis is a complex process that encompasses a series of tightly regulated sequential cellular events. Any interruptions in the process may result in CLP. As gene expression has temporal-spatial specificity during development, any abnormalities in gene expression at the palate development stages may cause CLP, which genetic studies cannot uncover. Thus, it is important to determine the expression profiles of genes in palate development. However, it is difficult to investigate the detailed human palate development process due to ethical considerations because human palate development stage occurs from 6–12 weeks of the embryonic period, making it important to use animal models to explore human palatal development. Although mice and chickens are the most conventional animal models to investigate the development of the palate [4,5,6], there are some limitations to using them, as their palates do not possess the same anatomical, morphological, and physiological features as humans, and the most obvious limitation is the developmental period. It is known that human palatogenesis proceeds from 6 to 12 weeks, which is in the early embryonic period, while in mice, this process occurs at the late-middle stage of mouse embryogenesis, on embryonic days (E) 12–15, relative to the 19–21-day embryonic phase.

**Figure 1:**
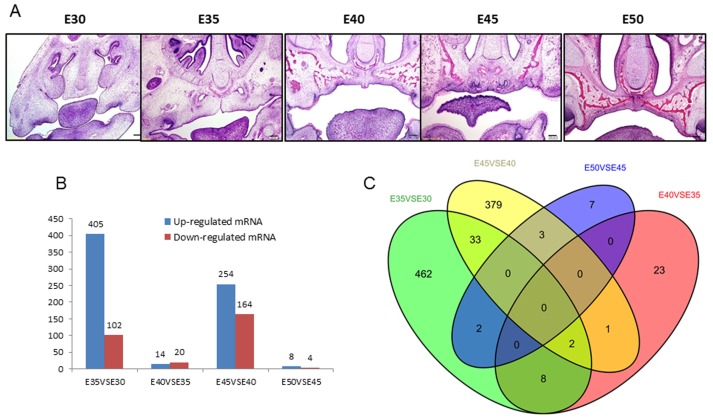
Gene expression profiles in secondary palate morphogenesis. (**A**) The palate development of miniature pigs from E30–50. Scale bar, 200 μm. (**B**) The differential expression genes between stages from E30–50. (**C**) Venn diagrams showing the numbers of transcriptional alterations between stages.

The miniature pig is similar to the human in terms of anatomy, histology, physiology, and nutrition metabolism in processes such as tooth development, which makes it an extensively used large animal model in biomedical research fields [7,8,9,10]. In our recent study, by performing a detailed histological analysis under light and electron microscopes, we characterized the palate developmental process of the miniature pig, which is more similar to humans than mice. We found that at E30, the primary (median) and secondary (lateral) palatal shelves, the primary structures of the palate appeared obviously and the secondary palatal shelves had initially grown vertically along the tongue initially. It was notable that E30–E35 was a rapid palate development stage. At this stage, the secondary palate shelves flipped horizontally and then fused to the opposite shelf. Most of the palate processes had joined at E40 until they were completely merged at E50, when obvious bone structure appeared. Then, the primitive form of the palate took shape (Figure 1A) [11,12,13,14]. The longer the palate development stage, the more detailed histological and genetic information was obtained due to the 114-day length of gestation in pigs compared with 19–21 days in mice. Additionally, analysis of the genome data of the Wuzhishan miniature pig was recently reported [15]. All of these factors make the miniature pig a valuable large animal model for investigating gene expression and functional regulation during palate development by RNA transcript sequencing.

As a widely used sequencing technology, high-throughput sequencing helps improve our knowledge of the molecular mechanisms underlying expression, regulation, and development by investigating the complete repertoire of expressed RNA transcripts in specific cells or tissues at specific stages of development [16]. Several microarray analyses of gene expression during murine palatogenesis were previously performed and they did not provide complete analyses of the gene expression patterns of palate development [17,18].

Here, we present differential gene expression data and analyze the gene regulatory networks of morphology at the key stages in palatal morphogenesis in a large animal model, the miniature pig, by RNA sequencing (RNA-Seq). Genes and pathways that were stage-specific and had differential regulation were identified. Furthermore, some interesting previously unreported pathways and genes such as major histocompatibility complex (MHC) class II -related genes and cardiac development-related genes were detected, which had stage-specific expressions during palate development. The results of our work on palate development in the miniature pig suggested deeper molecular mechanisms to explore in future research efforts, which could contribute to understanding the pathogenesis of CLP.

## 2. Results

### 2.1. Analysis of mRNA Sequencing in Different Stages of Sus scrofa Secondary Palate Development

To identify the mRNA expression during secondary palate development in miniature pigs, 15 cDNA libraries were constructed with total RNA from 5 different stages of palate development according to our previous study, and raw reads were generated by Illumina deep RNA sequencing (each stage had 3 cDNA libraries). Overviews of the sequencing and assembly results are shown in Table S1. Mapping results for the 15 libraries (5 developmental stages) are shown in Table S2. The comparison of adjacent developmental stages, E35 versus E30, E40VSE35, E45VSE40, and E50VSE45, detected 405 upregulated and 102 downregulated genes, 14 upregulated and 20 downregulated genes, 254 upregulated and 164 downregulated genes, and 8 upregulated and 4 downregulated genes, respectively (Figure 1B, Table S3). Venn diagrams show that among the 920 differentially expressed genes (DEGs) in the 5 stages, 10 genes were the same between E35-vs-E30 and E40-vs-E35, 35 genes were the same between E35-vs-E30 and E45-vs-E40, 2 genes were the same between E35-vs-E30 and E50-vs-E45, 3 genes were the same between E40-vs-E35 and E45-vs-E40, there were no overlapping DEGs between E40-vs-E35 and E50-vs-E45, 3 genes were the same between E45-vs-E40 and E50-vs-E45, only 2 genes, paired-box gene 9 (*PAX9*) and laminin subunit gamma 2 (*LAMC2*), were the same among E35-vs-E30, E40-vs-E35, and E45-vs-E40, and no overlapping DEGs were detected for the 4 or 3 other pairwise comparisons (Figure 1C, Table S3). Of the most significantly upregulated DEGs at stage E35-vs-E30, some were related to mineralization, such as bone sialoprotein 2 (*IBSP* or *BSP2*), dentin matrix acidic phosphoprotein 1 (*DMP1*), cartilage intermediate layer protein 2 (*CILP2*), and carbohydrate sulfotransferase 13 (*CHST13*). Additionally, at stage E40-vs-E35, the most significantly changed DEGs were associated with MHC class II protein complex and metabolic processes, such as *Sus scrofa* MHC class II histocompatibility antigen SLA-DQA (*SLA-DQA1*), cytochrome P450, family 26, subfamily B, and polypeptide 1 (*CYP26B1*). At E45-vs-E40, the most significantly changed DEGs were associated with ion binding, such as sentan (*SNTN*), and muscle-related genes were downregulated, such as tripartite motif-containing protein 72 (*TRIM72*), and UNC45B (*CMYA4*). Furthermore, at E50, the most significantly changed DEGs were related to ion binding, such as metallothionein-2A (*MT2A*) and carbonic anhydrase II (*CA2*) (Table S3).

**Figure 2:**
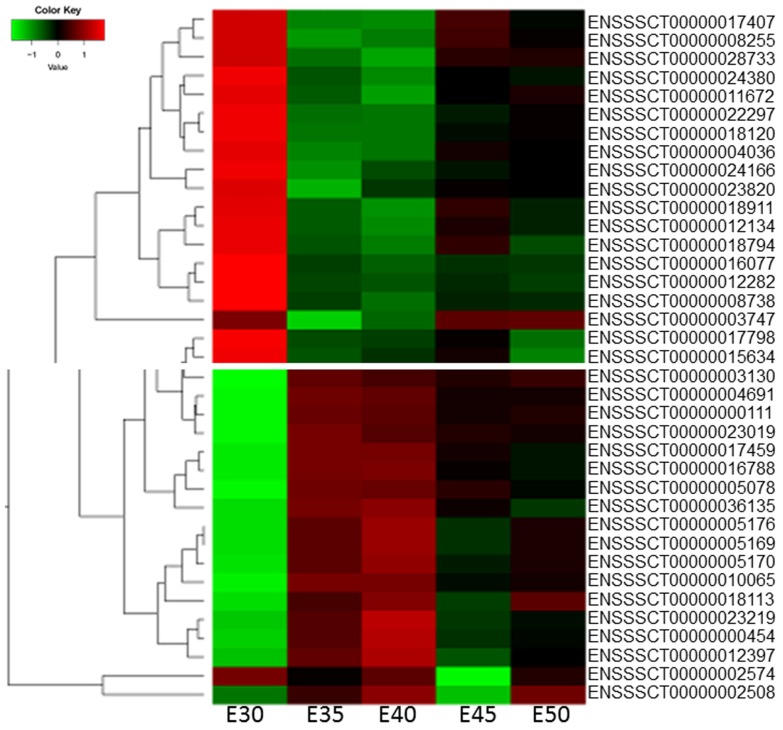
Hierarchical cluster analysis for differentially expressed genes (DEGs) showed different expression levels from stages E30 to E50.

We selected parts of the heatmaps of the five stages, which showed the differential expression of genes. We found that the gene expression profiles were the most different between E30 and E35, followed by E40 compared with E45, while the differential gene expression between E35 and E40 and E45 between E50 was small (Figure 2), in accordance with the Venn diagram (Figure 1B,C).

### 2.2. Gene Ontology Analysis of the Screened Genes in Five Developmental Stages of Palatogenesis

**Figure 3:**
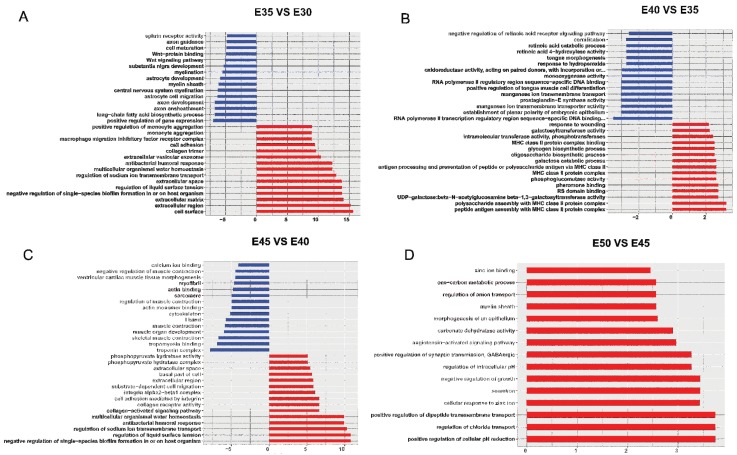
The significant gene ontology (GO) terms of differentially expressed genes during secondary palate development. (**A**) The significantly enriched GO terms between E35VSE30; (**B**) The significantly enriched GO terms between E40VSE35; (**C**) The significantly enriched GO terms between E45VSE40; (**D**) The significantly enriched GO terms between E50VSE45. The *y* axis showed the GO terms and the *x* axis showed the negative logarithm of the *p* value (−LgP). A larger −LgP indicated a smaller *p* value for the difference.

The significantly enriched gene ontology (GO) terms based on the 405 upregulated DEGs in E35 versus E30 were strongly correlated with cell surface (*HIP*, etc.), extracellular region (*IBSP*, etc.), regulation of liquid surface tension (BPI fold containing family A, member 1 (*BPIFA1*), etc.), regulation of sodium ion transmembrane transport (*BPIFA1*, etc.), collagen trimer (collagen, type V, alpha 1 (*COL5A1*), etc.), cell adhesion (*IBSP*, *COL5A1*, etc.), and face morphogenesis (*PAX9*, etc.). Additionally, the GO terms of the downregulated DEGs mainly involved long-chain fatty acid biosynthetic process (proteolipid protein 1 (*PLP1*), etc.) and the WNT (wingless-type mouse mammary tumor virus integration site family) signaling pathway (lymphoid enhancer-binding factor 1 (*LEF1*), etc.). Interestingly, many GO terms related to the nervous system, such as axon development (*PLP1*) and central nervous system myelination (myelin regulatory factor (*MYRF*), etc.), were found to be downregulated (Figure 3A, Table S4, Figure S1). The GO terms of significantly upregulated genes at E40 versus E35 were correlated strongly with MHC class II protein complex (*Sus scrofa* MHC class II DR-alpha (*SLA-DRA*), etc.) and glucose metabolic process (phosphoglucomutase-2 (*PGM2*), etc.), while the significantly downregulated differential genes for E40 versus E35 were mainly involved in tongue development (*CYP26B1*, etc.), odontogenesis (*PAX9*, etc.), retinoic acid binding (*CYP26B1*, etc.), RNA polymerase II regulatory region sequence-specific DNA binding (forkhead box F2 (*FOXF2*), etc.), and oxidoreductase activity (LOC110261221, etc.) (Figure 3B, Table S4, Figure S1). At E45VSE40, the significantly upregulated differential genes were focused on regulation of liquid surface tension (*BPIFA1*, etc.), collagen receptor activity (integrin, alpha 2 (*IGTA2*), etc.), and extracellular space (cilia and flagella associated protein 58 (*CFAP58*), etc.), while the significantly downregulated differential genes were associated with troponin complex (troponin C type 2 (*TNNC2*), etc.), muscle fiber development (myogenin (*MYOG*), etc.), cytoskeleton (myosin, heavy chain 7B, cardiac muscle, beta (*MYH7B*), etc.), and calcium ion binding (delta-like 1 homolog (*DLK1*), etc.) (Figure 3C, Table S4, Figure S1). Interestingly, at E50VSE45, the significantly differentially expressed genes with GO terms were all upregulated, focusing on regulation of chloride transport (*CA2*), positive regulation of cellular pH reduction (CA2), and zinc ion binding (*MT2A*, etc.) (Figure 3D, Table S4, Figure S1).

### 2.3. Kyoto Encyclopedia of Genes and Genomes (KEGG) Analysis of the Screened Genes in Five Stages of Palatogenesis

**Figure 4:**
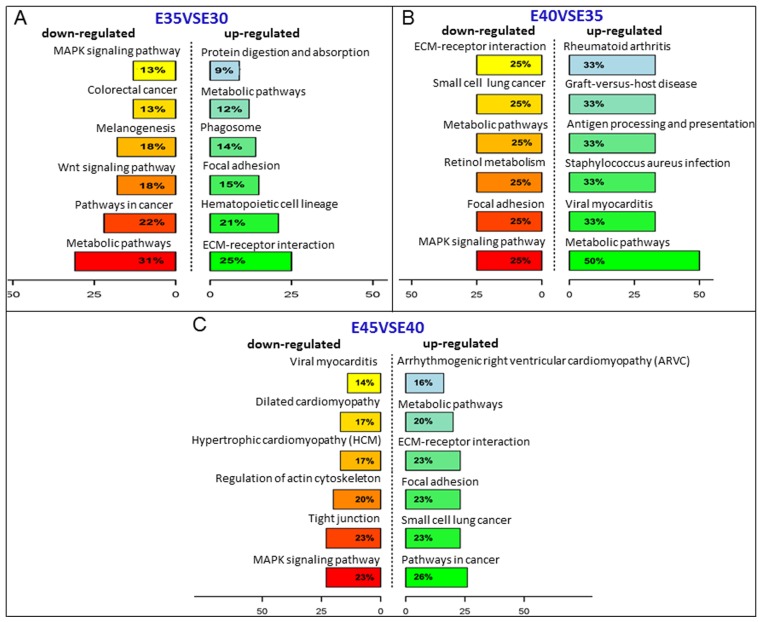
The significantly up- and down-regulated pathways during secondary palatal development. (**A**) The significant up- and down-regulated pathways between E35VSE30; (**B**) The significant up- and down-regulated pathways between E40VSE35; (**C**) The significant up- and down-regulated pathways between E45VSE40. The *y* axis shows the pathways and the *x* axis shows the rich factor (DEGs in the pathway/total DEGs).

A total of 41, 7, 19, and 1 DEGs involved in 16, 20, 12, and 1 pathways were predicted in the pairwise comparisons of E35VSE30, E40VSE35, E45VSE40, and E50VSE45, respectively (*p* ≤ 0.5), by aligning to the KEGG database, and gene function categories were analyzed with emphasis on biochemical pathways. In E35VSE30, the extracellular matrix (ECM)-receptor interaction pathway (ko04512) containing the most DEGs was upregulated, which regulated cellular activities such as adhesion, migration, differentiation, proliferation, and apoptosis (directly or indirectly). The WNT signaling pathway and MAPK signaling pathway were downregulated (Figure 4A, Table S5, Figure S2). Among those significantly upregulated genes, IBSP (BSP2), 1 of the top 10 upregulated DEGs, with roles in many GO terms such as extracellular region, cell adhesion, biomineral tissue development, etc., and a member of ko04510 (Focal adhesion) and ko04512 (ECM-receptor interaction), was detected as significantly upregulated in E35 compared with E30. The encoded protein binds tightly to hydroxyapatite, appears to form an integral part of the mineralized matrix, and is probably important for cell-matrix interactions and promotes Arg-Gly-Asp-dependent cell attachment [19]. For E40VSE35, 19 different pathways were screened, and only 7 DEGs were found, with approximately 2–3 DEGs in 1 pathway. In these pathways, most DEGs were *Sus scrofa* MHC class II-related genes such as *SLA-DQA1* and *SLA-DRA*, which was also a component of lysosomal membrane, cell surface, and extracellular vesicular exosome according to its GO terms. Retinol metabolism (ko00830), involved in palate development in previous reports, and a member of this pathway, *CYP26B1*, was detected as downregulated at this stage (Figure 4B, Table S5, Figure S2) [1,2,20,21,22,23]. In E45VSE40, interestingly, some DEGs were associated with heart development, such as *Sus scrofa* actin, alpha, cardiac muscle 1 (*ACTC1*), *Sus scrofa* myosin light chain 2V (*MLC2V*), *Sus scrofa* myosin, heavy chain 7, cardiac muscle, beta (*MYH7*), and cell structures as tight junction and focal adhesion. The regulation of actin cytoskeleton (ko05412), focal adhesion (ko04510), and subcellular structures that modulate the regulatory effects (i.e., signaling events) of a cell in response to ECM adhesion, and MAPK signaling (ko04010) contained 1/3 of DEGs, and the HH signaling pathway was also screened (Figure 4C, Table S5, Figure S2). Furthermore, in E50, only one DEG (carbonic anhydrase II in Nitrogen metabolism pathway) was upregulated relative to E45, which was associated with the GO terms apical part of cell, extracellular vesicular exosome, ion binding, etc. (Table S5, Figure S2).

**Figure 5:**
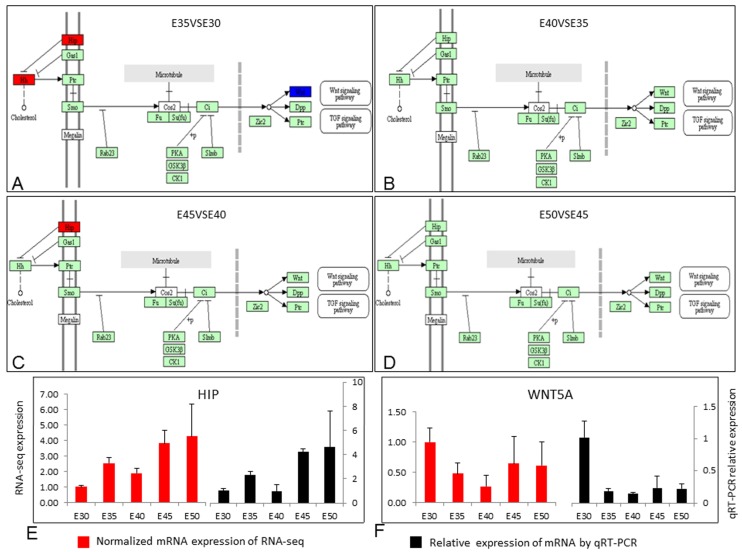
The different expression of HH (hedgehog) signaling. (**A**–**D**) Hedgehog signaling pathway (ko04340) expression between E35 and E30 (**A**); E40 VS E35 (**B**); E45 and E40 (**C**); E50 VS E45 (**D**). (**E**) Real-time reverse transcription-polymerase chain reaction (qRT-PCR) of *HIP* from E30–50. (**F**) qRT-PCR of *WNT5A* from E30–50.

We also investigated several signals that played important roles during secondary palate development showed dynamic changes in the five stages. These signals included retinol metabolism (ko00830), MAPK signaling (ko04010), WNT signaling (ko04310), and the HH signaling pathway (ko04340) (Figure 4, Figure S2, Figure 5A–D) [1,2,5,6,17,18,21,22,23]. Real-time reverse transcription-polymerase chain reaction (qRT-PCR) of *HIP* and *WNT5A* was performed and confirmed the variation of HH signaling (Figure 5E–F).

### 2.4. Quantitative Analysis of mRNA Expression

**Figure 6:**
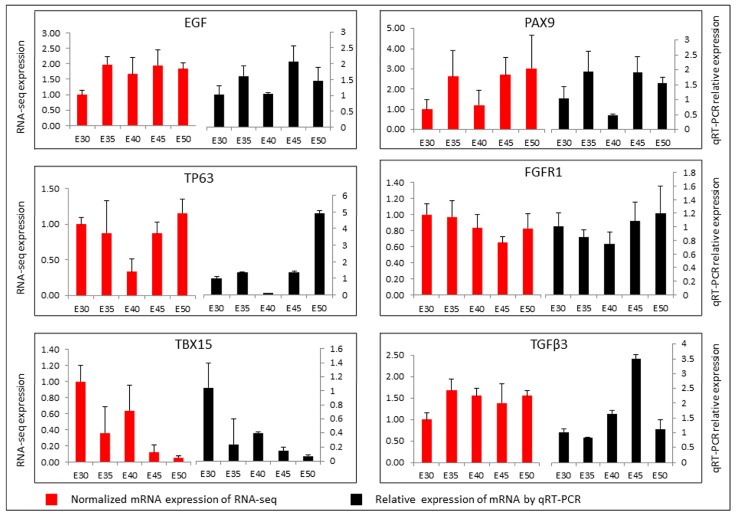
Expressions of the six mRNAs using RNA-Seq and qRT-PCR. All the data are shown as mean ± SE.

The validation of expression profiles assessed from the quantity of sequence data captured was performed by qRT-PCR. We selected eight genes associated with cleft palate (i.e., fibroblast growth factor receptor (*FGFR*), epidermal growth factor (*EGF*), *PAX9*, and tumor protein p63 (*TP63*)) [24,25,26,27,28,29,30]. With the exception of transforming growth factor β3 (*TGFβ3*), the expression patterns of the selected mRNAs were in accordance with those determined using high-throughput sequencing, which verified that the sequencing data in the present study were reliable and could be subjected to further analysis (Figure 6). For genes, there are different isoforms at the mRNA level. For example, there are three transcript variants of *TGFβ3* in *Sus scrofa* in Genbank. In the RNA-seq results, there were two isoforms of *TGFβ3*. For real-time RT-PCR detection, we selected the common sequence of *TGFβ3* mRNA and one sequence of a *TGFβ3* isoform to compare by RNA-seq. The presence of isoforms might influence the alignment of the RNA-seq and qRT-PCR results. 

### 2.5. The Interaction Network of Differentially Expressed Genes and Pathways (Signal-Gene Net)

**Figure 7:**
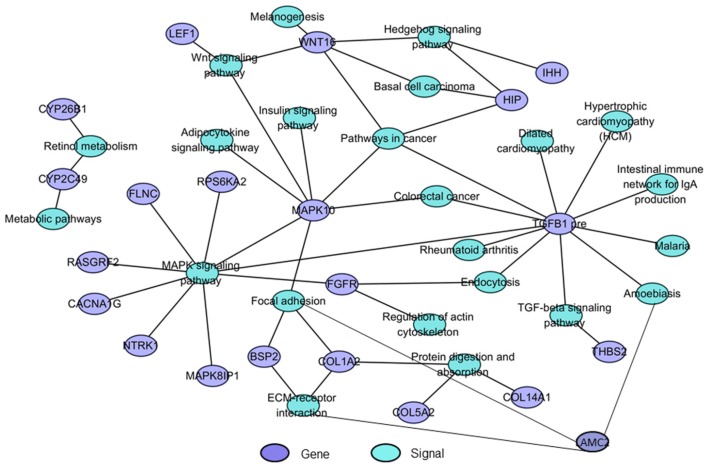
Signal-gene interaction network. Significant DEGs and signals playing important roles during secondary palate development were selected and analyzed by the Signal-Gene construction. Purple cycle nodes represent genes, blue cycle nodes represent signals. Lines between two different color nodes represent interactions between signals and genes. The more lines of a signal, the more genes it involves, and vice versa.

To further elucidate the relationship between the differentially expressed genes and signals that played important roles during secondary palate development, a signal-gene net based on KEGG analysis was constructed (Figure 7). Twenty-one significant DEGs involving 23 pathways were selected as the potential targets during palate development (Table S6). As shown in Figure 7, the WNT, HH, MAPK, TGFβ, and retinol metabolism signaling pathways, which may play key roles in craniofacial morphogenesis as previously reported [1,2,20,21], and *TGFβ1* precursor, *MAPK10*, *WNT16*, *FGFR*, and *HIP* were involved in many pathway functions, suggesting new clues regarding key genes in these signaling pathways during palate development.

## 3. Discussion

In general, there are two major ways to investigate the mechanisms of congenital fetus malformation: genetics and development. With a genetics approach, pathogenic genes are screened and their functions are identified in development. Most genetic mutations of monogenetic diseases are detected by this method. For polygenetic diseases, such as CLP, there are many factors influencing the incidence of disease. It is important to investigate pathogenesis through development, and the detailed normal developmental process of organ development is essential, including both the anatomical and molecular biology fields. The mammalian palate is important because it separates the nasal and oral cavities, facilitating breathing, pronunciation, and deglutition. It is divided into two parts anatomically: The primary palatal processes and secondary palatal processes. Elaborate cellular differentiation and dynamic morphogenesis are involved in palatogenesis [11]. This process is artificially divided into four approximate stages according to the secondary palate morphogenesis: The vertical growth stage with the bilateral palatal shelves growing downward vertically along the tongue; the elevating stage with the palatal shelves raising up to the horizontal position above the tongue; the fusion stage with the bilateral shelves fusing with one other and fusing forward with the primary palate processes; and the maturity stage with the initial palate forming [11,12,13,14]. If these events are disrupted, CLP may take place. To conduct a more detailed investigation, we subdivided the fusion stage into the early fusion and late fusion stage in miniature pigs, as most CLP occurs in the fusing stage [2,6,14]. Genome information about normal palate development is limited, while most genome analyses of palate development have focused on the genetics of CLP [30,31,32,33]. Mice were used as models, with differential gene expression identified for only some pathways, such as the TGFβ and WNT signaling pathways, which were previously a focus [17,18]. Since the primary palate has a distinct developmental origin from the secondary palate, and the development of the secondary palate serves as a paradigm for embryonic development in general, as virtually all of the molecular processes and signaling pathways associated with normal palatal ontogeny are mirrored in the embryogenesis of multiple other systems, the palatogenesis is focus on the secondary palate [1,2,17,18,34]. With the rapid development of high-throughput sequencing technologies, large-scale molecular datasets have been obtained, and new bioinformatics tools such as KEGG and GO analysis are used to understand the high-level functions and utilities of biological systems, which can detect more information about genome sequences. According to the genome analysis of miniature pigs, we selected mRNA sequences to obtain more information on palate development. 

In our previous study on palate development, we confirmed the application of the miniature pig as a large animal model [14]. As E30–35 was the fastest growth period of miniature pig palate development and showed the maximum morphological change, it was a key period in miniature pig palate development. For this reason, at this stage, the maximum quantity of DEGs was detected, and most of them focused on functions related to cell structures via GO terms such as extracellular region, cell surface, proteinaceous extracellular matrix, cell adhesion, and collagen trimer. KEGG analysis was in accordance with GO terms. At this stage, the ECM-receptor interaction pathway (ko04512), which contained the maximum quantity of DEGs, took roles in regulating cellular activities such as proliferation, apoptosis, migration, differentiation, and adhesion (directly or indirectly). Interestingly, we found that at this stage, some DEGs associated with mineralization, such as IBSP (BSP2) and DMP1, were upregulated. At this stage, bone differentiation may have occurred in the palate (Figure 1A). Another interesting result was that DEGs associated with MHC class II-related genes were upregulated from E35 to E40, which was not reported previously. At this stage, the shelves raise up to a horizontal position above the tongue, move close to each other, and contact in the midline. It was found that a multilayer epithelial seam, called medial edge epithelium (MEE), formed when palate shelves fused to the opposing epithelia, which covered the tips of the palatal shelves. Then, MEE cells died or transformed into mesenchyme and the dead cells were phagocytosed by macrophages [14,35]. For this reason, MHC class II-related genes, which are mainly expressed on macrophages, may have been upregulated. Another interesting change was that at this stage, DEGs associated with tongue development were downregulated. In our recent study, the tongue development of miniature pigs was in accordance with the palate in that after E35, the tongue dropped rapidly to the mouth floor, then tongue development slowed down, and the muscle fibers of the tongue began to mature [36]. Thus, the DEGs associated with tongue development were downregulated. It has been shown that retinoic acid (RA) plays important roles during palate development, but excess RA causes cleft palate in the fetuses of both rodents and humans. The coordinated regulation of retinoid metabolism is essential for normal palatogenesis. CYP26B1 is a key player in normal palatogenesis, as the endogenous RA level is determined by the balance of RA-synthesizing (retinaldehyde dehydrogenases: RALDHs) and RA-degrading enzymes (CYP26s). It remains unknown whether RA exhibits a gradient distribution and functions as a morphogen in the developing palate. Our findings may provide a clue that in the early fusion stage (E35–40), RA promotes the fusing of the palate shelves and facilitates the growth of the maxillary and palatine bones, as *CYP26B1* was downregulated [37].

From E40 to E45, we detected some DEGs that were associated with heart development. The literature has reported that some genes are involved in both cardiac and craniofacial malformations, such as homeobox B1 (*HOXB1*) and Meis homeobox 2 (*MEIS2*) [38,39]. *Hoxb1* mutant mice displayed altered transcription effectors of developmental signaling pathways (bone morphogenetic protein (Bmp), sonic hedgehog (Shh), vascular endothelial growth factor A (Vegfa)), and transcription factors (Paired-box gene 3 (*Pax3*), SRY (sex determining region Y)-box transcription factor 9 (*Sox9*)) [38]. In *Meis2*-null mouse embryos, severely abnormal developing cartilage was observed in the palate, and 67% of mutants also presented malformed palates, though this was less severe [39]. At approximately E50, organogenesis of the swine heart can be observed [40], and we speculated that this stage was also an important stage for the heart development of miniature pigs, and some genes might influence both the heart and palate. During this stage, the palate shelves fuse, and bone formation proceeds. Potentially for this reason, we observed that some genes associated with cilia and flagella, such as *SNTN*, cilia and flagella associated protein 58 (*CFAP58*), were upregulated. Emerging evidence has shown that loss or malfunction of primary cilia or ciliary proteins in bone and cartilage is associated with developmental and functional defects [41]. Tissue-specific conditional loss of ciliary genes with different functions produces profoundly different facial phenotypes, including cleft palate [42]. Another interesting result was that the number of DEGs in the late fusion stage (E45VSE40) (418 DEGs) was greater than that in the early fusion stage (E40VSE35) (34 DEGs), which shows that the late fusion stage needs more attention in CLP pathogenesis. The palate was almost formed, and there was little change from E45 to E50, with the minimum quantity of DEGs and only one DEG, *CA2*, upregulated by KEGG at this stage. *CA2* expressed high levels in bone-resorbing cells, osteoclasts, during bone resorption. Blocking the expression of *CA2* led to decreased bone resorption [43]. 

From these data, it was shown that the secondary palate development of miniature pigs is a complex and elaborate process. As an important process in palate formation, bone regulation was dynamically remodeled via promotion from E30–45 and resorption at E50. 

Signaling networks regulate palate development by a complex series of context-dependent morphogenetic signaling events. Many genes involved in palatogenesis have been discovered through the use of genetically manipulated mouse or chicken models as well as human genetic studies, especially the SHH, TGFβ, WNT, MAPK, BMP, FGF, and retinol metabolism pathways, etc. [1,2,5,6,17,18,20,21,22,23,24,25,26,27,28,29,30,44,45,46,47]. Similar to these studies, we investigated whether several signaling pathways important in mouse and chicken palate development were also observed to show significant dynamic expression profiles in the five stages of miniature pig palatogenesis, such as retinol metabolism, MAPK signaling, WNT signaling, and HH signaling pathway. These results further verified the roles of these signaling pathways in palatogenesis. Recently, connective tissue growth factor (CTGF) or CCN2 was shown to play an essential role in the formation of the secondary palate by interacting with a number of other factors that are important for palate development, including bone morphogenetic proteins (BMPs), fibroblast growth factors (FGFs), epidermal growth factor (EGF), WNT proteins, and transforming growth factor-s (TGF-s) [45]. To further analyze the relationship between DEGs and these signals in palatogenesis and screen promising target genes such as CTGF, we constructed signal-gene net patterns based on KEGG analysis to obtain a comprehensive evaluation of these differentially expressed genes. We detected some genes in these pathways that had not previously been associated with palate development, such as *HIP*, *MAPK10*, and *WNT16*, which showed they may be promising targets during palate development. It is also notable that two DEGs (*PAX9* and *LAMC2*) were the same among E35-vs-E30, E40-vs-E35, and E45-vs-E40. *PAX9* was reported to regulate a molecular network involving BMP4, FGF10, SHH signaling, and odd-skipped related transcription factor 2 (OSR2) to regulate the palate development [47]. *Pax9*-deficient mice had a cleft secondary palate [29]. All of these data showed that *PAX9* plays important roles in palate development. The other DEG was *LAMC2*, which is involved in basement membrane assembly, positive regulation of cell migration and proliferation, system development, and is a key component of hemidesmosomes that connect keratinocytes to the underlying dermis. Mice with a targeted deletion of *Lamc2* (laminin γ2) die within a few days after birth, presumably due to skin blistering (dehydration) [48]. Due to its roles in the basement membrane zone, we speculated that this gene might play roles during epithelial breakdown and palate fusion and, as such, is a novel target to study palate pathogenesis. These results suggested that further work might focus on these genes as promising candidate genes in palate development and CLP pathogenesis.

## 4. Materials and Methods 

All animals and experiments were conducted under the “Guidelines for Experimental Animals” of the Ministry of Science and Technology (Beijing, China). Animal suffering was minimized. Experimental procedures involving miniature pigs were approved by the Animal Care and Use Committee of Capital Medical University, Beijing, China, under permit No. CMU-B20140412 on 12 April 2014.

### 4.1. Experimental Animals, Tissues Collection and Histological Staining

We obtained pregnant Wuzhishan miniature pigs from the Institute of Animal Science of the Chinese Agriculture University. Animals were allowed access to food and water ad libitum under normal conditions and were humanely sacrificed as necessary to ameliorate suffering. In brief, pregnant sows were anesthetized with a combination of 6 mg/kg ketamine chloride and 0.6 mg/kg xylazine, and pregnancy and the fetal state were roughly determined by B-mode ultrasonography. Secondary (lateral) palate processes representing vertical, horizontal, early fusion, late fusion, and the end of the fusion stages corresponding to the five key stages (E30, E35, E40, E45, E50) of palate development in miniature pigs were selected and dissected according to our previous research [14]. At least three miniature pigs were used for each time point. Thus, each stage possessed three biological replicates. The morphological stages of the palate at E30, E35, E40, E45, and E50 were verified by serial histological sections.

### 4.2. Total RNA Isolation, cDNA Libraries Construction and Sequencing

Total RNA was isolated from the secondary palate samples at the five stages using an RNA purification kit (Invitrogen, CA, USA) according to the Invitrogen protocol. The quantity and purity of the total RNA were analyzed by a Bioanalyzer 2100 and RNA 6000 Nano LabChip Kit (Agilent, CA, USA). At each stage, three digital gene expression profiling libraries were constructed from three different palatal samples with RNAs whose integrity numbers (RIN) were above 7.0. According to the manufacturer’s protocol (Illumina, CA, USA), approximately 10 μg of total RNA was used to generate cDNA. Then, single-end sequencing was performed on an Illumina Hiseq 2500 sequencer at LC Sciences (Hangzhou, China) following the manufacturer’s protocol as previously reported [49]. 

### 4.3. Gene Expression Profiles of Different Development Stages 

After removing adaptor sequences, ambiguous ‘N’ nucleotides (with a ratio of ‘N’ greater than 5%) and low-quality sequences (with quality scores less than 20) were filtered, and the remaining clean reads were then subjected to quality assessment, which was critically evaluated by LC Sciences (Hangzhou, China). DEGs between adjacent stages were identified with *p* value ≤ 0.05 and log2-fold-change (log2FC) ≥ 1 based on expression levels. GO terms (http://www.geneontology.org/) and KEGG pathway (http://www.genome.jp/kegg/) analyses of DEGs were conducted to investigate significant functions and pathways of genes. 

### 4.4. Gene Expression Validation 

To verify the sequencing data, the expression patterns of eight genes (*HIP*, *WNT5A*, *EGF*, *PAX9*, *TP63*, *FGFR1*, *TBX15*, and *TGFβ3*) over the five stages were selected via qRT-PCR. We synthesized cDNA from 2-µg aliquots of RNA, random hexamers or oligo(dT), and reverse transcriptase, according to the manufacturer’s protocol (Invitrogen, CA, USA). Real-time PCR was performed with the QuantiTect SYBR Green PCR Kit (Qiagen, Duesseldorf, Germany) and an Icycler iQ Multicolor Real-time PCR Detection System as described in previous work [50]. qRT-PCR experiments were performed in triplicate and at least three times for each gene. Changes in gene expression were determined by the 2^−∆∆*C*t^ method. The primers used for specific genes are shown in Table S7.

## 5. Conclusions

In summary, our results provide fundamental molecular resources for the five developmental stages of the secondary palate, from emerging to complete formation. A total of 920 DEGs were identified and presented a molecular basis for studying palate development. Some core candidate DEGs and pathways were indicated here, which might be of particular interest in further molecular mechanism studies of palate development in miniature pigs. These DEGs could be used to increase the understanding of molecular processes that affect palate development and the etiology of cleft palate.

## Supplementary Materials

Supplementary materials can be found at https://www.mdpi.com/1422-0067/20/17/4284/s1. Table S1. Summary of sequence data generated for miniature pig secondary palate development transcriptome, and quality filtering. Table S2. Alignment results of 15 libraries mapping to the reference transcriptome. Table S3. All differentially expressed genes with *p* ≤ 0.05 compared with the other palate development stages. Table S4. The genes involving significant GO groups (*p* ≤ 0.05) in E30–50. Table S5. The genes involving significant pathways (*p* ≤ 0.05) in E30–50. Table S6. The genes and pathways identified by signal-gene analysis. Table S7. Primer sequences used in real-time RT-PCR. Figure S1. The statistics of GO enrichment in E30–50. Figure S2. The statistics of pathway enrichment in E30–50.

## Abbreviations

CLP	cleft lip/palate
E	embryonic days
EMT	epithelial-mesenchymal transformation
VS	versus
MEE	medial edge epithelium
DEGs	different expression genes
GO	Gene ontology
KEGG	Kyoto encyclopedia of genesand genomes
qRT-PCR	Real-time reverse transcription-polymerase chain reaction
EDTA	Ethylene Diamine Tetraacetic Acid
HE	hematoxylin and eosin
