# Supplementary material for: Dynamic mRNA Expression Analysis of the Secondary Palatal Morphogenesis in Miniature Pigs
Source: Int J Mol Sci. 2019 Sep 1;20(17):4284. doi: 10.3390/ijms20174284 (PMC6747431; doi:10.3390/ijms20174284)
Supplement: Supplementary file 1 [file ijms-20-04284-s001.zip › supple/Table S1.docx]

**Table S1 Summary of sequence data generated for minipig secondary palate development transcriptome, and quality filtering**

| Samples | Raw Data | | Q20% | | Q30% | GC% |
| --- | --- | --- | --- | --- | --- | --- |
|  | Read | Valid | |  |  |  |
| 1-E30 | 1.3E+07 | 1.3E+07 | | 99.15 | 96.67 | 49.16 |
| 2-E30 | 1.2E+07 | 1.2E+07 | | 99.15 | 96.68 | 49.62 |
| 3-E30 | 1E+07 | 1E+07 | | 99.18 | 96.81 | 47.76 |
| 4-E35 | 1.4E+07 | 1.4E+07 | | 99.13 | 96.58 | 50.20 |
| 5-E35 | 1.1E+07 | 1.1E+07 | | 99.14 | 96.62 | 50.19 |
| 6-E35 | 1.2E+07 | 1.2E+07 | | 99.16 | 96.71 | 49.08 |
| 7-E40 | 1.1E+07 | 1.1E+07 | | 99.17 | 96.77 | 48.33 |
| 8-E40 | 1.1E+07 | 1.1E+07 | | 99.13 | 96.62 | 49.56 |
| 9-E40 | 1.1E+07 | 1.1E+07 | | 99.14 | 96.70 | 47.87 |
| 10-E45 | 1.1E+07 | 1.1E+07 | | 99.15 | 96.76 | 47.26 |
| 11-E45 | 9781488 | 9745201 | | 99.17 | 96.79 | 47.72 |
| 12-E45 | 1E+07 | 1E+07 | | 99.16 | 96.74 | 48.28 |
| 13-E50 | 9836283 | 9798994 | | 99.18 | 96.80 | 47.39 |
| 14-E50 | 1.1E+07 | 1.1E+07 | | 99.22 | 96.93 | 47.86 |
| 15-E50 | 1.1E+07 | 1.1E+07 | | 99.15 | 96.77 | 47.14 |
