# Supplementary material for: Dynamic mRNA Expression Analysis of the Secondary Palatal Morphogenesis in Miniature Pigs
Source: Int J Mol Sci. 2019 Sep 1;20(17):4284. doi: 10.3390/ijms20174284 (PMC6747431; doi:10.3390/ijms20174284)
Supplement: Supplementary file 1 [file ijms-20-04284-s001.zip › supple/Table S2.docx]

**Table S2 Alignment results of 15 libraries mapping to the reference transcriptome**

| **Sample** | **Total valid reads** | **Unique valid reads** | **Total mapped reads** | **Unique match** | **Total Unique gene** | **Expression gene** |
| --- | --- | --- | --- | --- | --- | --- |
| 1-E30 | 12,920,986 | 6,681,416 | 8,654,342 | 4,019,955 | 27370 | 23042 |
| 2-E30 | 11,594,920 | 6,300,180 | 7,787,744 | 3,988,056 | 27370 | 22883 |
| 3-E30 | 10,289,600 | 5,165,836 | 7,141,219 | 3,241,000 | 27370 | 22407 |
| 4-E35 | 13,580,867 | 6,674,821 | 9,100,273 | 4,197,196 | 27370 | 23207 |
| 5-E35 | 11,321,203 | 5,847,449 | 7,767,540 | 3,802,358 | 27370 | 22799 |
| 6-E35 | 11,583,227 | 6,235,089 | 7,629,188 | 3,914,876 | 27370 | 22918 |
| 7-E40 | 11,311,957 | 5,482,360 | 7,819,913 | 3,478,410 | 27370 | 23465 |
| 8-E40 | 10,787,108 | 5,593,585 | 7,306,264 | 3,521,132 | 27370 | 22987 |
| 9-E40 | 10,516,628 | 5,854,467 | 6,828,052 | 3,572,894 | 27370 | 22993 |
| 10-E45 | 10,732,224 | 5,777,114 | 7,160,385 | 3,579,101 | 27370 | 23194 |
| 11-E45 | 9,745,201 | 5,007,860 | 6,550,420 | 3,118,973 | 27370 | 22724 |
| 12-E45 | 10,042,491 | 5,139,668 | 6,793,904 | 3,215,928 | 27370 | 23009 |
| 13-E50 | 9,798,994 | 5,364,612 | 6,651,011 | 3,352,817 | 27370 | 22833 |
| 14-E50 | 10,812,487 | 5,387,991 | 7,399,718 | 3,333,869 | 27370 | 23110 |
| 15-E50 | 10,558,394 | 5,572,233 | 7,021,661 | 3,345,670 | 27370 | 23172 |
