# Supplementary material for: Dynamic mRNA Expression Analysis of the Secondary Palatal Morphogenesis in Miniature Pigs
Source: Int J Mol Sci. 2019 Sep 1;20(17):4284. doi: 10.3390/ijms20174284 (PMC6747431; doi:10.3390/ijms20174284)
Supplement: Supplementary file 1 [file ijms-20-04284-s001.zip › supple/Table S7.docx]

**Table S7 Primer sequences used in real-time PCR**

| **Gene symbol** | **Forward Primer(5’-3’)** | **Reversed Primer(5’-3’)** |
| --- | --- | --- |
| HIP | AACAACTCCCGTTACACCGAC | GCCCGAGGAACGAGATAAAGT |
| WNT5A | TAGTCCACGCAAACCCTTTC | TCACTCGGTCTTTGGGAACT |
| EGF | CATTTGGACGGTGTCTTGAGTT | GCATTATCTTGACGGGCTTG |
| PAX9 | TCCCAGACTGTGCTGTCGTTC | GGCGGCTCCGTCTATCACTC |
| P63 | CCAGCACCGCCAAGTCAG | GGGGCACCGTTTCACCAC |
| FGFR1 | TGCGACAGAGGAACAGGGAG | GGAGCAGACAAAACCAACACC |
| TBX 15 | CAGCAAGGAGGCAGCACAG | GGATGGCGATAAAAGATGAGAA |
| TGFB3 | TCGGTGCTGGGGCTGTA | AGGTGAGGTCTGTTGCTTTTGT |
| GAPDH | TACGAGATGCCTGATGGTCAGGTCA | TGGAGTTGTACGTGGCCTCATGGAC |
