# Supplementary figures and images for: Dynamic mRNA Expression Analysis of the Secondary Palatal Morphogenesis in Miniature Pigs
Source: Int J Mol Sci. 2019 Sep 1;20(17):4284. doi: 10.3390/ijms20174284 (PMC6747431; doi:10.3390/ijms20174284)

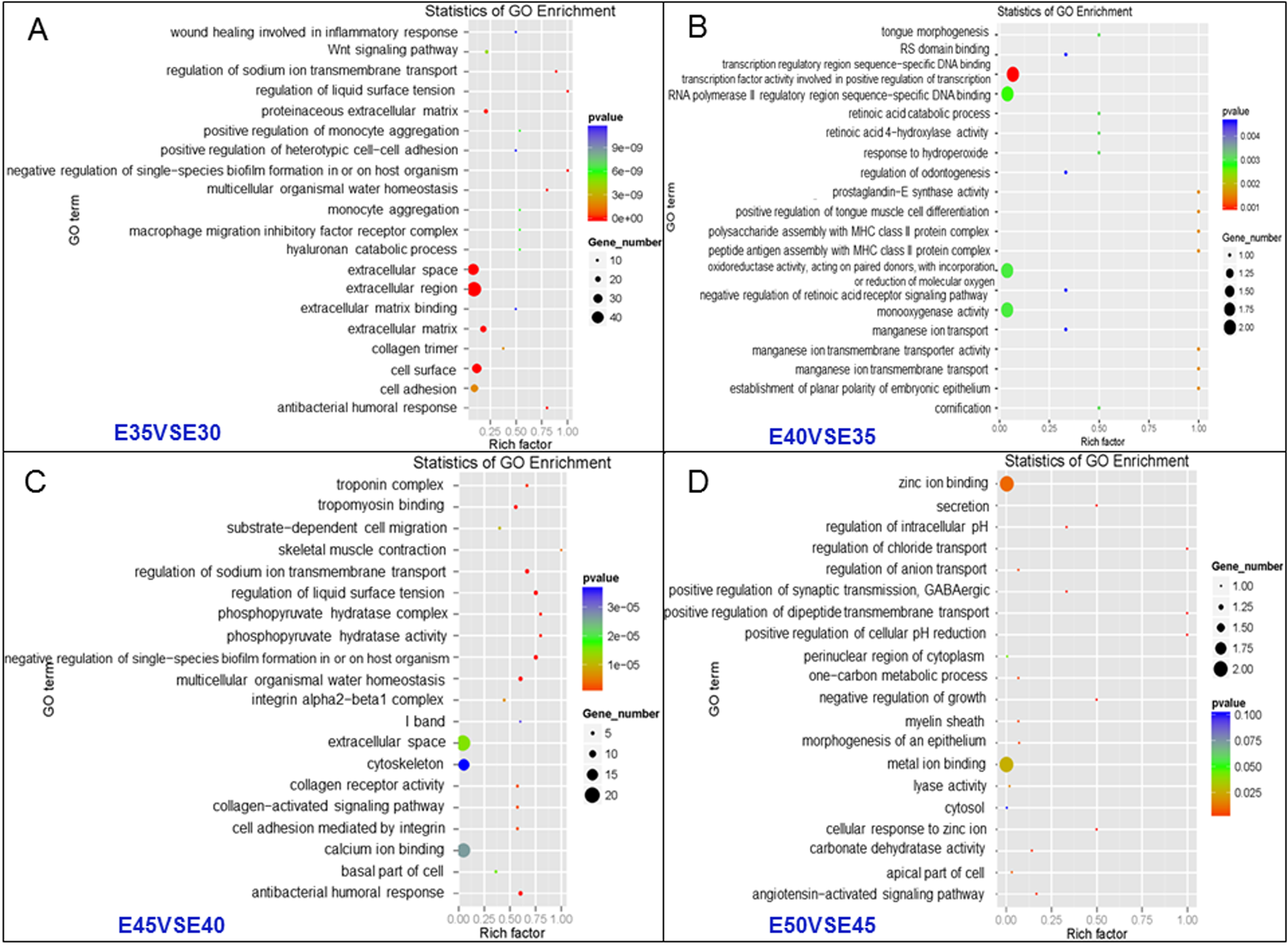

Supplement: Supplementary file 1 [file ijms-20-04284-s001.zip › supple/Fig.S1-GO.tif]

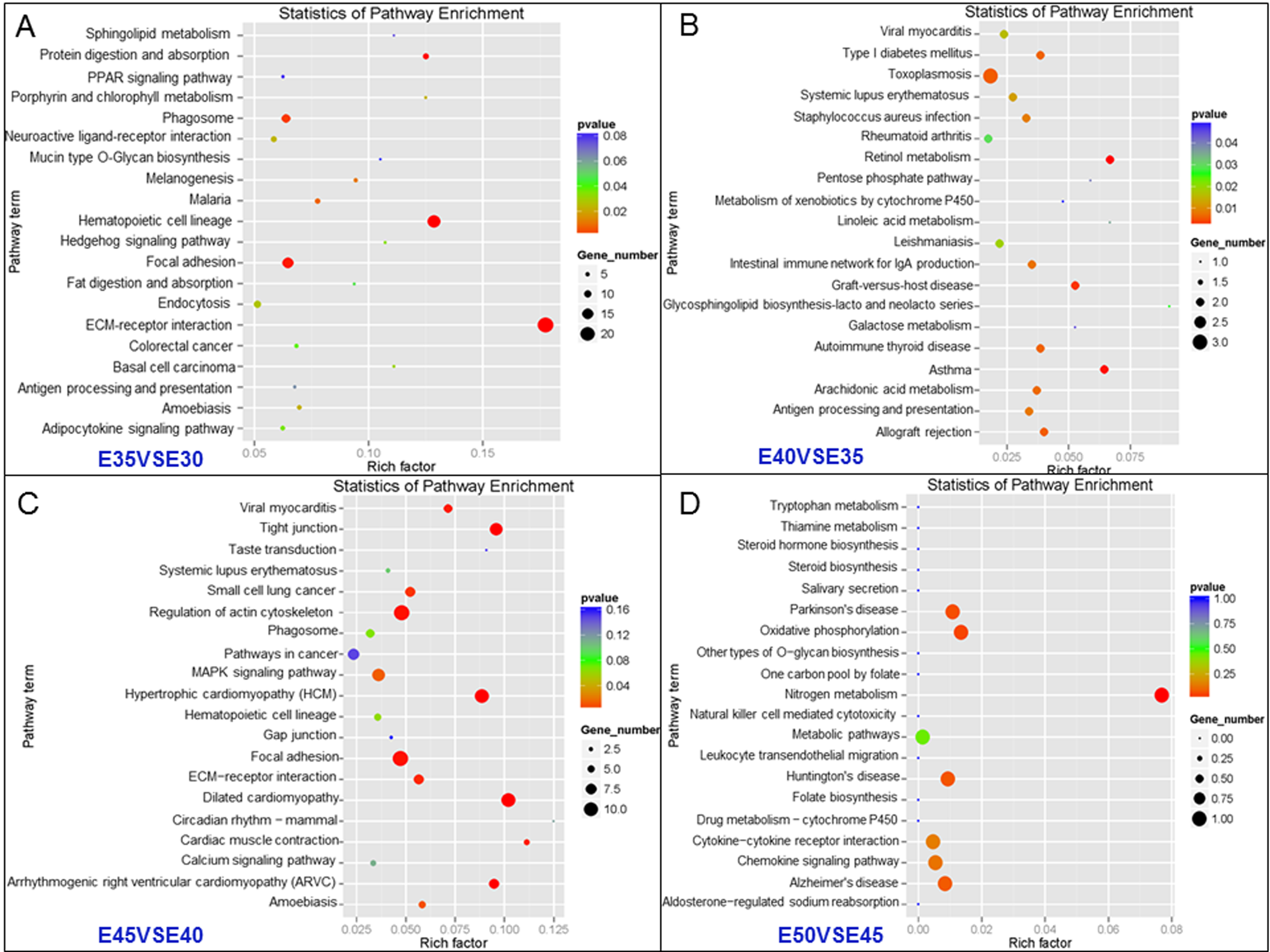

Supplement: Supplementary file 1 [file ijms-20-04284-s001.zip › supple/Fig.S2-KEGG.tif]
